# The genome of the sparganosis tapeworm *Spirometra erinaceieuropaei* isolated from the biopsy of a migrating brain lesion

**DOI:** 10.1186/s13059-014-0510-3

**Published:** 2014-11-21

**Authors:** Hayley M Bennett, Hoi Ping Mok, Effrossyni Gkrania-Klotsas, Isheng J Tsai, Eleanor J Stanley, Nagui M Antoun, Avril Coghlan, Bhavana Harsha, Alessandra Traini, Diogo M Ribeiro, Sascha Steinbiss, Sebastian B Lucas, Kieren SJ Allinson, Stephen J Price, Thomas S Santarius, Andrew J Carmichael, Peter L Chiodini, Nancy Holroyd, Andrew F Dean, Matthew Berriman

**Affiliations:** Wellcome Trust Sanger Institute, Parasite Genomics, Cambridge, CB10 1SA UK; Department of Histopathology Section, Addenbrookes’s NHS Trust, Cambridge, CB2 0QQ UK; Department of Infectious Diseases, Addenbrooke’s NHS Trust, Cambridge, CB2 0QQ UK; Department of Radiology, Addenbrookes’s NHS Trust, Cambridge, CB2 0QQ UK; Department of Neurosurgery, Addenbrookes’s NHS Trust, Cambridge, CB2 0QQ UK; Hospital for Tropical Diseases and London School of Hygiene and Tropical Medicine, London, WC1E 6JD UK; Department of Histopathology, St Thomas’s Hospital, London, SE1 UK; Biodiversity Research Center, Academia Sinica, Taipei, 11529 Taiwan; Eagle Genomics, Babraham Research Campus, Babraham, Cambridge, CB22 3AT UK

## Abstract

**Background:**

Sparganosis is an infection with a larval Diphyllobothriidea tapeworm. From a rare cerebral case presented at a clinic in the UK, DNA was recovered from a biopsy sample and used to determine the causative species as *Spirometra erinaceieuropaei* through sequencing of the *cox1* gene. From the same DNA, we have produced a draft genome, the first of its kind for this species, and used it to perform a comparative genomics analysis and to investigate known and potential tapeworm drug targets in this tapeworm.

**Results:**

The 1.26 Gb draft genome of *S. erinaceieuropaei* is currently the largest reported for any flatworm. Through investigation of β-tubulin genes, we predict that *S. erinaceieuropaei* larvae are insensitive to the tapeworm drug albendazole. We find that many putative tapeworm drug targets are also present in *S. erinaceieuropaei*, allowing possible cross application of new drugs. In comparison to other sequenced tapeworm species we observe expansion of protease classes, and of Kuntiz-type protease inhibitors. Expanded gene families in this tapeworm also include those that are involved in processes that add post-translational diversity to the protein landscape, intracellular transport, transcriptional regulation and detoxification.

**Conclusions:**

The *S. erinaceieuropaei* genome begins to give us insight into an order of tapeworms previously uncharacterized at the genome-wide level. From a single clinical case we have begun to sketch a picture of the characteristics of these organisms. Finally, our work represents a significant technological achievement as we present a draft genome sequence of a rare tapeworm, and from a small amount of starting material.

**Electronic supplementary material:**

The online version of this article (doi:10.1186/s13059-014-0510-3) contains supplementary material, which is available to authorized users.

## Background

Tapeworms affect the lives of millions worldwide. Of those, the debilitating or potentially deadly cysticercosis and echinococcosis are priority targets for the World Health Organization [1]. The availability of genomes of the major disease-causing species *Echinococcus* spp. and *Taenia solium* have heralded the way for increased research progress and new venues for intervention [2,3]. However, molecular knowledge regarding rarer tapeworm infections, such as those with *Spirometra erinaceieuropaei*, is scarce.

**Figure 1:**
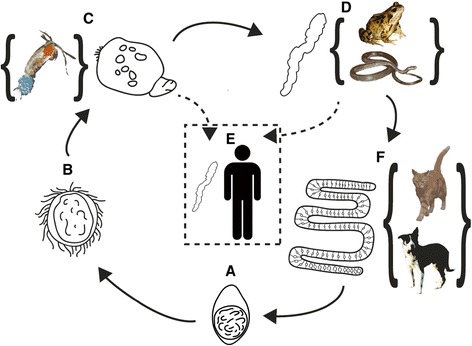
**Life cycle of** ***Spirometra erinaceieuropaei.*** **(A)** Unembryonated eggs are released and embryonate over 8 to 14 days in water [10]. **(B,C)** Eggs hatch to release free-swimming coracidia **(B)**, which parasitize copepods (such as *Cyclops* sp.) and develop into procercoid larvae **(C)**. **(D)** On ingestion of the copepod by a veterbrate host - such as a tadpole, frog or snake - these develop into plerocercoid larvae, also known as sparganum. The plerocercoid larvae reside in the tissues of these organisms. The larval stage infection can be passed on when the host organism is eaten. **(E)** Humans become infected by ingestion of a live larva, or in some cases direct contact, such as a poultice of infected frog tissue on the eye. A larva can also infect humans when an infected copepod is ingested. **(F)** The larva only develops into the adult form in the gastrointestinal tract once it reaches a definitive host, such as a cat or a dog, where eggs are passed in the faeces (A). Curly brackets denote known hosts, though the full extent of the possible hosts and life cycle complexity of this tapeworm species have not been well characterized. Images of *S. erinaceieuropaei* are guided by the experimental life history photographed by Lee *et al*. [10]. Source of modified images; snake [11]; frog courtesy of Anant Patel MD; cyclops [12] (Matt Wilson/Jay Clark, NOAA NMFS AFSC); dog [13] (Richard New Forest).

Compared with more common human-infective tapeworms, *S. erinaceieuropaei* has an even more complex life cycle (Figure 1) involving a minimum of three hosts for completion. *Spirometra* spp. are found worldwide but human infections are most often reported in Asian countries, typically China, South Korea, Japan and Thailand, although several recent travel and migration-related cases of sparganosis have occurred in Europe [4,5]. The infective stage for humans is a motile, secondary larval form known as the sparganum. Infection can occur through the ingestion of raw tadpoles, the consumption of undercooked frogs or snakes, or the use of frog meat as a poultice on open wounds or eyes [6]. However, infections are also thought to arise through accidental ingestion of infected copepods from contaminated drinking water or from swallowing water whilst swimming [6,7]. Once the larva is inside the human body, its final location appears unrestricted - reported sites of infection include the eyes, subcutaneous tissue, abdominal cavity, spinal cord and brain [6,8]. Pathology is associated with location; for example, infections in the brain can cause convulsions or paralysis. The worm is usually only discovered during exploratory surgery and treated by its subsequent removal [4,9].

Infections with *S. erinaceieuropaei* and closely related tapeworms are rare in humans. Pampiglione *et al.* [7] collated 300 cases worldwide between 1953 and 2003. A review of Chinese language articles revealed more cases, over 1,000 in Mainland China since 1882 [6]. Because these infections occur rarely, clinicians are not likely to consider this diagnosis until many other tests have been performed, and usually the worm is only discovered during surgery. Infections are even more unexpected in Europe, as there were only seven reported cases in the literature before 2003 [7]. Recent cases of travel- or migration-related infection in Europe have occurred in the last three years [4,5].

In this study we describe genome sequencing of a single parasite isolated from a 50-year-old male patient who presented in the East of England with a debilitating larval tapeworm infection that showed migration across the brain over a 4-year period. By PCR on DNA extracted from a biopsy sample, we identified the worm as *S. erinaceieuropaei*, distinguishing it from *S. proliferum*, a taxonomically related species known for its ability to proliferate (with potentially fatal consequences) in the human host. From a histological section, we isolated the parasite and produced a draft genome sequence. We examined the known targets of drugs in the parasite genome and used this to predict how this parasite would have responded to chemotherapy-based treatments. From a large-scale comparison of gene families across the tapeworms, we identified gene family expansions in this cestode, which is the first of its order (Diphyllobothriidea) whose genome has been sequenced. These data contribute to the growing global database for identifying parasites and parasite provenance and will serve as a resource for identifying new treatments for sparganosis.

## Results

### Migrating cerebral lesions indicate sparganosis

A 50-year-old man of Chinese ethnicity was admitted for investigation of symptoms that included headaches, complex partial and tonic-clonic seizures, reported episodes of altered smell and flashback of memory and memory impairment as well as progressive right-sided pain. The patient had lived in the UK for 20 years but visited his homeland often. MRI of the brain revealed an abnormality in the right medial temporal lobe of high signal on T2 (oedema) with a cluster of ring-enhancing lesions (Additional file 1). The diagnostic possibilities were of an inflammatory or a neoplastic lesion.

The patient tested negative for HIV, tuberculosis, lime disease, syphilis, coccidioides, histoplasma and cryptococcus. A cysticercus immunoblot with patient serum was negative. Inflammatory screens for antinuclear and anti-neutrophil antibodies and complement (C3 and C4) were normal and the patient was systemically well. C-reactive protein (CRP) level was within the normal range (3 mg/L), as was the erythrocyte sedimentation rate (6 mm/h). Computed tomography of his chest abdomen and pelvis showed no abnormality.

Right temporal lobe neurosurgical biopsy showed a mixed lymphocytic (B and T cells) non-necrotising, non-granulomatous inflammation with a few plasma cells. Tuberculosis was suspected but no organisms visualised.

**Figure 2:**
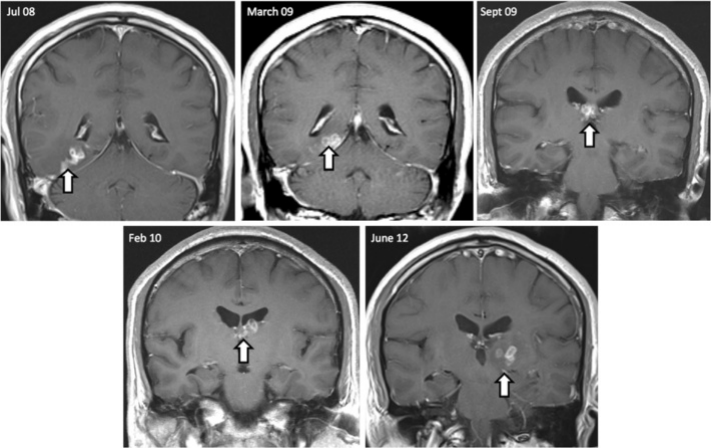
**Sequential imaging over a 4-year period identifies migrating lesions.** Sequential imaging over 4-year period: July 2008 to June 2012. All images are coronal T1 scans post gadolinium. The shifting white arrow, from right to left hemispheres, depicts the migration pattern of a cluster of ring-enhancing lesions.

**Figure 3:**
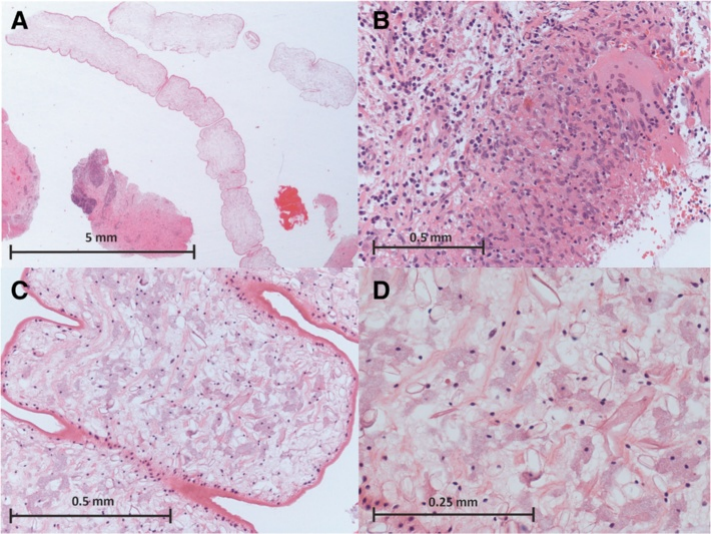
**Morphological examination of biopsy reveals infection is sparganosis. (A)** A 1.6-fold magnified view of the worm and adjacent brain tissue from biopsy; the worm is unsegmented (although there are infoldings of the cuticle), without intestine, and uniform in internal structure. **(B)** A host granulomatous reaction featuring focal necrosis, epithelioid and multi-nucleated giant cells of macrophage-derivation, some plasma cells and lymphocytes but no eosinophils that, considered in isolation, resembles tuberculosis (×20). **(C)** A 20-fold magnified view of the worm demonstrates the eosinophilic syncytial tegument, sub-tegumental nuclear layer, and the internal watery stroma that includes thin muscle fibres, round cells, and ‘empty’ tubular excretory ducts. **(D)** A 40-fold magnified view of the internal stroma exhibits thin eosinophilic muscle fibres and stromal cells with pale haematoxyphilic cytoplasm. All images stained with haematoxylin and eosin and scale bars are 5 mm **(A)**, 0.5 mm **(B,C)** and 0.25 mm **(D)**.

A series of MRI images in the ensuing four years demonstrated contralateral gradual migration of the multiloculate lesions from the right hemisphere through the thalamus (Figure 2). Throughout the disease process, the lesion had moved at least 5 cm through the brain. A second biopsy, from the left thalamus, showed granulomatous inflammation, focal necrosis and an approximately 1 cm ribbon-shaped cestode larval worm without mouthparts or hooklets. With the pathognominic morphology of a sparganum, it was so diagnosed at the Department of Histopathology, St Thomas’ Hospital, and the Department of Clinical Parasitology, Hospital for Tropical Diseases (Figure 3). Immediately post-operation, the patient was given albendazole and is now systemically well.

### Molecular identification of the causative agent as *S. erinaceieuropaei*

**Figure 4:**
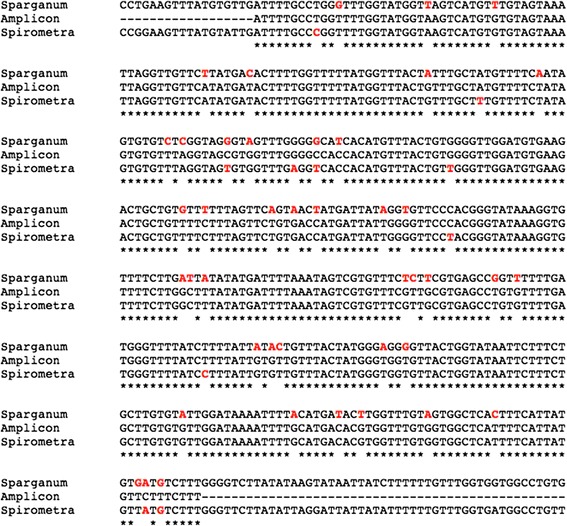
**Alignment of** ***cox1*** **amplicon with** ***cox1*** **sequence from** ***S. erinaceieuropaei*** **and** ***S. proliferum.*** Consensus sequence from forward and reverse capillary reads of *cox1* amplicon (line name = amplicon) aligned against the two species *S. erinaceieuropaei* (line name = Spirometra) and *S. proliferum* (line name = Sparganum). Bases highlighted in red differ from the amplicon; asterisks indicate consensus between all sequences.

DNA was extracted from the formalin-fixed paraffin embedded worm and PCR and Sanger capillary sequencing was carried using primers for cytochrome oxidase c 1 (*cox1*), the mitochondrial gene often referred to as ‘the barcode of life’. A consensus sequence from forward and reverse reads was used to search against the EMBL database using BLASTN, and returned *cox1* from *S. erinaceieuropaei* as a top hit, notably higher than the search result against the proliferative *S. proliferum*, which is morphologically similar but would have a poor prognosis for the patient. Alignment of the sequences confirmed this finding (Figure 4). The sequence shared 98% identity with *S. erinaceieuropaei* compared with 90% identity with *S. proliferum*.

No exact *cox1* match was found in *S. erinaceieuropaei* isolates that had previously been sequenced. However, the base anomalies to previously known *S. erinaceieuropaei cox1* sequence were subsequently confirmed in whole genome data (Additional file 2).

Interestingly, consensus sequence from two further mitochondrial genes, *nad1* and *cox3*, were identical to *S. erinaceieuropaei* sequences from isolates collected from frogs in Hunan province, China [14].

### The genome of *S. erinaceieuropaei*

Using 0.048 μg DNA isolated from a formalin-fixed biopsy, a 1.26 Gb draft assembly of the *S. erinaceieuropaei* genome was assembled from two lanes of paired-end Illumina HiSeq 2000. Protein-coding genes were predicted using the software MAKER [15], which used the gene prediction software Augustus [16], GeneMark [17] and SNAP [5] alongside species-specific gene models from *Caenorhabditis elegans* and Cestodes as evidence. Genome statistics are presented in Table 1 and genome quality assessment in the Materials and methods section.

**Table 1:** **Genome-wide statistics for the** ***S. erinaceieuropaei*** **assembly and gene predictions**

**Genome statistics**	
Size of genome (Gb)	1.26
GC content (%)	46
Number of scaffolds	483,112
N50	4,647
Largest scaffold (bp)	89,810
Number of predicted genes	39,856
Gene density per Mb	31.6
Length of proteome (amino acids)	6,844,057
Maximum protein length (amino acids)	1,947
Average protein length (amino acids)	172
Average exon length (bp)	259
Median exon length (bp)	213
Average exons per transcript	2
Median exons per transcript	2
Total length of contained introns (Mb)	41
Average intron length (bp)	1,065
Median intron length (bp)	418
CEGMA	73/248
BLAST output against CEGMA	231/248

To assess the completeness of the genome, we used the Core Eukaryotic Genes Mapping Approach (CEGMA) software [14], which includes hidden Markov models for 458 core eukaryotic genes. A subset of these, 248 genes, are extremely highly conserved and are believed to be present in virtually all eukaryotes as single copy genes. The proportion of this subset that can be mapped into a target genome provides an assessment of the completeness of the genome. The standard CEGMA pipeline identified 73 of the 248 core CEGMA genes (29.44%) in the assembly as complete, with an additional 115 core CEGMA genes reported as partially contained (46.7%). The average number of predictions for each complete gene was 1.42 (1.81 for partial genes), indicating some level of expansion of the assembly due to its draft nature. Analysing the raw BLAST output file produced by CEGMA revealed that 93.1% of all 458 CEGMA genes had significant BLAST matches with e-values of <1e-05 (88.2% in predicted gene models). The fragmented nature of the assembly had therefore prevented many genes from meeting the more stringent matching criteria set by CEGMA. The BLAST results suggest that most of the core genes are identifiable in the genome but that many genes are present as fragments within the assembly.

Using RepeatModeller [18] and RepeatMasker [19], 43% (537 Mb) of the *S. erinaceieuropaei* genome was masked as repetitive, including 16% long interspersed elements (LINEs), 4% short interspersed elements (SINEs), 2% long terminal repeat (LTR) elements and 19% unclassified repetitive elements.

We interrogated the *S. erinaceieuropaei* genome with a recently published EST data set [20] and found that all 5,641 ESTs had a significant BLAST match with e-values of <1e-05, indicating that the genome contains useful molecular data. Additionally, we found that 73% of ESTs were within predicted gene models.

### The characteristics of the current tapeworm chemotherapy targets in *S. erinaceieuropaei*

We focused our initial interrogation of the genome on features with the highest potential clinical relevance, such as targets of tapeworm chemotherapy. β-Tubulin is a microtubule component targeted by the benzimidazole class of drugs, such as albendazole, a commonly used drug for tapeworm infection. In the roundworm *Haemonchus contortus*, well-characterized mutations, namely phenylalanine to tyrosine at codon 167 and 200, are known to confer resistance to benzimidazoles in both the laboratory and field studies [21,22]. Searching for β-tubulin genes by TBLASTX, using known *Echinococcus multilocularis* sequences, revealed potential homologs in the *S. erinaceieuropaei* genome. We aligned protein sequences with the region of interest, and found that one had tyrosine residues in the positions known to confer benzimidazole resistance (SPER_0000685601). A reciprocal BLAST search confirmed that the latter gene is a likely orthologue of *tub-2*, highly expressed in *E. multilocularis* larva. We also searched for β-tubulin transcripts by BLAST in recently published EST data from the larval stage of *S. erinaceieuropaei* [20], and found that of 26 β-tubulin ESTs, 24 contained the benzimidazole resistance-associated amino acids.

The drug praziquantel is also used to treat tapeworm infections [23]. Schistosomes, which are from another major clade of parasitic flatworms, are also sensitive to praziquantel and the calcium channel subunit CaV2 B has been postulated as the drug’s target [24]. In the case of schistosomes, the accessory β2a calcium channel subunit lacks two serine residues (likely phosphorylation sites for protein kinase C) that are conserved in vertebrate orthologues. When these residues are removed from rat β2a subunits by mutagenesis, reconstituted calcium channels become sensitive to praziquantel *in vitro* [25]*.* Although, there is still uncertainty about the exact target(s) of praziquantel, CaV2 B is the current best lead; we therefore examined the sequence characteristics of CaV2 B in *S. erinaceieuropaei.* To identify candidates, we searched using the sequences of genes encoding calcium channels from the *E. multilocularis* genome. The latter genes are long with many exons and long stretches of intronic sequence. Therefore, considering the fragmented nature of the *S. erinaceieuropaei* assembly, obtaining primarily partial BLAST matches from our gene transcripts for these genes was to be expected. Two out of four partial hits (SPER_0001175301 and SPER_0001441801) had an aligned region covering the phosphorylation residues identified as potential drug response modulators (225; 235 in rat β2a), and these contained a ‘sensitive’ asparagine and an alanine in the equivalent positions. The other two hits were shorter and encoded a threonine and a serine in these positions.

ATP-binding cassette (ABC) transporter proteins are efflux pumps that have relevance to multidrug resistance in nematodes and schistosomes [26]. A total number of 19 six-transmembrane helix ABC transporter domains (InterPro:IPR001140, Pfam: PF00664) were detected in *E. multilocularis* predicted gene transcripts, whereas a total of 37 of these domains were present in *S. erinaceieuropaei* predicted transcripts.

### New tapeworm drug targets in *S. erinaceieuropaei*

Our next approach concentrated on finding orthologues of putative tapeworm drug targets proposed during analysis of the *E. multilocularis* genome [2], many of which are targets of known cancer drugs, thus opening the door to a possible drug repurposing strategy for identifying new leads for development. Predicted gene transcripts from the assembled *S. erinaceieuropaei* genome were searched using TBLASTX for evidence of homologs of these targets (Table 2). There were significant hits for each putative target. Genes notable for both their high identity and completeness when aligned to the *E. multilocularis* sequences were adenine nucleotide translocator (SPER_0000599901), ribonucleoside diphosphate reductase (SPER_0000698501), calmodulin (SPER_0000219201), FK506 binding protein (SPER_0000627901) and elongation factor 2 (SPER_0001150701).

**Table 2:** **Putative tapeworm drug targets for which there is a TBLASTX hit in predicted** ***S. erinaceieuropaei*** **gene transcript (E-value >1e-10)**

**Putative** ***E. multilocularis*** **drug targets** ^**a**^	**TBLASTX E-value**	**Percentage completeness** ^**b**^	**Percentage identity of match**	***S. erinaceieuropaei*** **gene ID**
Thioredoxin glutathione reductase (TGR)	8e-77	28	73	SPER_0002850001
Fatty acid amide hydrolase	3e-59	39	50	SPER_0002366401
Adenine nucleotide translocator	e-161	79	84	SPER_0000599901
Inosine 5′ monophosphate dehyrogenase	2e-81	42	79	SPER_0001958401
Succinate semialdehyde dehyrogenase	1e-54	25	68	SPER_0002970001
Ribonucleoside diphosphate reductase	e-149	67	83	SPER_0000698501
Casein kinase II	e-114	47	93	SPER_0000626801
Hypoxanthine guanine phosphoribosyltransferase	2e-35	57	43	SPER_0000257601
Glycogen synthase kinase 3	2e-69	50	84	SPER_0001364001
Proteasome subunit	4e-21	36	59	SPER_0002270001
Calmodulin	1e-94	100	98	SPER_0000219201
FK506 binding protein	3e-43	100	70	SPER_0000627901
UMP-CMP kinase	6e-24	40	73	SPER_0000808401
Na+/K+ ATPase	0	40	91	SPER_0000981501
Carbonic anhydrase II	3e-39	84	60	SPER_0002854501
NADH dehydrogenase subunit 1	3e-22	55	62	SPER_0000882501
Translocator protein	1e-26	95	44	SPER_0002949701
Elongation factor 2	0	81	70	SPER_0001150701
Cathepsin B (cysteine protease)	6e-93	62	61	SPER_0002586301
Dual-specificity mitogen activated protein	3e-54	27	86	SPER_0000571801
Purine nucleoside phosphorylase	3e-77	69	61	SPER_0000360401

^a^Expressed in *E. multilocularis* larvae. ^b^Percentage of *E. multilocularis* genes covered by alignment with *S. erinaceieuropaei* sequence.

### Genes predicted to be involved in host-parasite interactions

We identified the gene encoding plerocercoid growth factor (PGF), also known as *S. erinaceieuropaei* cysteine protease (SeCP; SPER_002801201), thought to have a role in multiple aspects of host-parasite interaction [27,28]. PGF has previously been identified as the component of *Spirometra* species secretory products that binds to human growth factor receptors, stimulating growth [27]. It has been shown to coat the plerocercoid larval tegument of *Spirometra mansonoides* and has cysteine protease activity against collagen, perhaps enabling the parasite to digest host tissue during migration [29]. Reported PGF cleavage activity against immunoglobulin may also enable the parasite to moderate inflammation [30].

**Figure 5:**
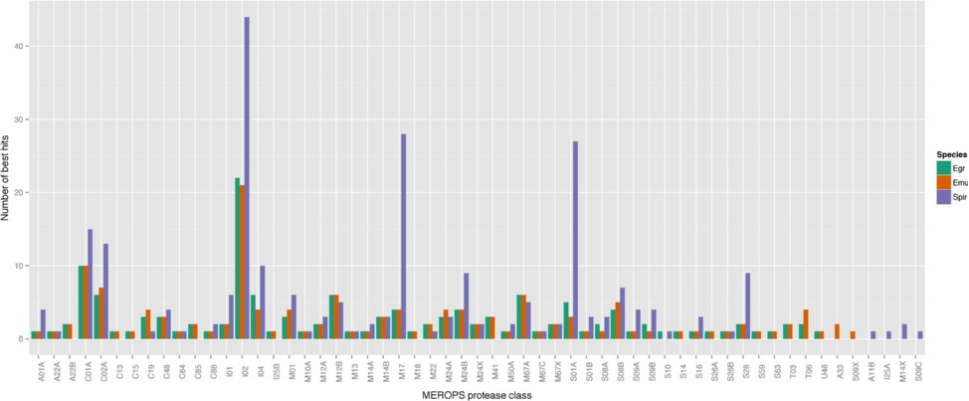
**Cross-species comparison of protease and protease inhibitor classes.** Protease and protease inhibitors by MEROPS classification in *Echinococcus granulosus* (green), *E. multilocularis* (orange) and *S. erinaceieuropaei* (purple) ordered alphabetically. In all species there is a large number of I02 class members, representing Kunitz-type protease inhibitors. The M17 class consists of leucyl aminopeptidases and the SO1A and S28 classes are serine endopeptidases.

Proteases and protease inhibitors are well known for their importance in host-parasite relationships [31-33]. Using InterProScan 5 we identified 302 sequences that contained predicted proteases or protease inhibitor domains. Using the MEROPS databases of proteases and protease inhibitors [34], we classified 242 of these genes and found the most abundant to be inhibitors of serine proteases (Figure 5). Interestingly, two classes of proteases appeared to be considerably expanded in comparison to *Echinococcus* spp.: both the M17 (amino-terminal leucyl aminopeptidases) and the serine endopeptidase classes S1A (chymotrypsin A-like) and S28 (lysosomal Pro-Xaa carboxypeptidase-like).

There is also an expanded family of nine M17 proteases in *Drosophila*, found to be highly expressed in sperm, though their exact functional role is unknown [35]. In the MEROPS resource *Drosophila persimilis* has the most abundant representation of the M17 family with 16 paralogues. In *S. erinaceieuropaei* we identified 28 putative M17 family proteases, 21 of which have clearly indicated active sites identified in the MEROPS analysis. Kunitz-type protease inhibitors (class I02) were notable for their abundance in all tapeworm species, and twice as many were detected in *S. erinaceieuropaei*.

Fatty acid transporters that bind low density lipoprotein (CD36 class B scavenger receptors) have been identified in other tapeworm genomes [2]. A TBLASTX search of the *S. erinaceieuropaei* transcripts using the *E. multilocularis* CD36 class B scavenger receptor (SCARB) sequences returned 14 hits. These transcripts gave reciprocal BLAST hits in the *E. multilocularis* genome, closest to the SCARB1.2, SCARB1.3 and SCARB2 genes. Thus, it appears that *Spirometra*, similar to other tapeworms, scavenges lipids from its host.

### Comparison of gene families in *S. erinaceieuropaei* with other characterized tapeworms

Previously, no tapeworm of this order of Cestoda (Diphyllobothriidea), which also includes the *Diphyllobothrium* genus responsible for diphyllobothriasis in humans, has been subject to whole genome sequencing. Therefore, this genome represents the first opportunity to investigate the genetic differences to the more characterized Cyclophyllidea tapeworms (for example, *Taenia* spp. and *Echinococcus* spp.).

**Figure 6:**
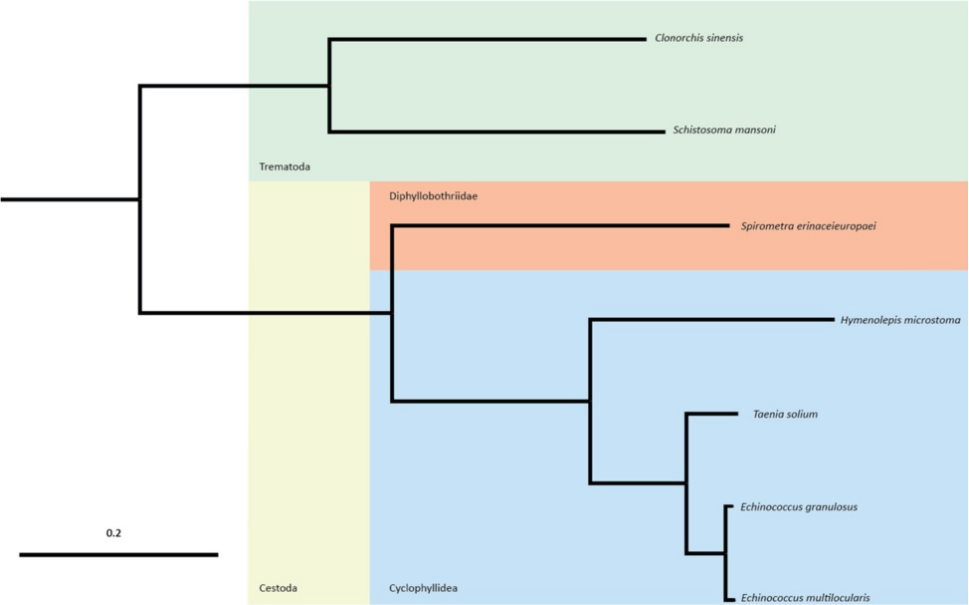
**Phylogeny of cestodes demonstrating the relationship of** ***S. erinaceieuropaei*** **to the Cyclophyllidea species.** Phylogenetic tree of all platyhelminth ComparaEnsembl GeneTree species outrooted by *Capitella teleta* and *Crassostrea gigas*. All orthologues of gene families (protein fasta files) from Compara were filtered to include representatives from at least seven species, and these were aligned with multiple alignment program for amino acid or nucleotide sequences (MAFFT). Poor alignments were filtered out using GBlocks and the remaining concatenated to PHYLIP multiple alignment format for passing to raxmlHPC along with the partition model. raxmlHPC was run with random seed 2131. Scale bar represents length of horizontal branch corresponding to a rate of genetic change per base of 0.2.

To identify genes that have duplicated or been lost in *S. erinaceieuropaei* we used the ComparaEnsembl GeneTrees pipeline to identify gene families across the following tapeworm genomes: *E. multilocularis*, *Echinococcus granulosus*, *T. solium* and *Hymenolepis microstoma*. Genomes from the trematodes *Schistosoma mansoni* and *Clonorchis sinensis* were also included in the analysis, along with outgroup genomes from *Capitella teleta* (a marine polychaete worm) and *Crassostrea gigas* (pacific oyster). For details of each tree see Additional file 3. A genome-wide phylogeny based on genes shared between all seven species fitted expected phylogenic relationships (Figure 6).

Given the fragmentary nature of the *S. erinaceieuropaei* genome, there was potential for the apparent number of predicted genes per family to be inflated by fragments from the same gene appearing more than once in the same family. There was indeed some indication that this was the case when gene families were ranked by the ratio of the number of *S. erinaceieuropaei* to *E. multilocularis* genes (Additional file 4); the highest apparently expanded protein family was titin, the largest known natural protein, and therefore a potential source for a huge number of alignable fragments. *Unc-22* (twitchin), a giant intracellular protein, was also apparent in the top of the list. The distribution of the median length of predicted proteins encoded by each gene family indicated that the *S. erinaceieuropaei* gene predictions were short compared with the other cestode species (Additional file 5). A plot of *E. multilocularis* median protein lengths against the number of *S. erinaceieuropaei* proteins in the same family confirmed this trend (Additional file 6).

To get a more accurate estimation of gene family expansions, potentially representing specialization or adaptation within the *Spirometra* lineage, we ranked gene families by the ratio of the total cumulative length of encoded *S. erinaceieuropaei* proteins to the cumulative length of the corresponding *E. multilocularis* proteins. A ratio cutoff of 3 was used to define the most expanded families, and to avoid apparent duplications that could be caused by divergent haplotypes within the assembly. There were 83 gene families that matched these criteria and the putative function of each family was investigated (Additional file 7). The M17 protease class identified in our previous MEROPS analysis was confirmed by our expansion criteria (ranked 21).

We investigated the total protein length of gene families that had previously been described as expanded in tapeworm species (Table 3) [2]. Expansion of tetraspanin is not apparent in *S. erinaceieuropaei*, demonstrating that there are differences between the evolutionary history of these proteins between Diphyllobothriidea and Cyclophyllidea tapeworm orders. Based on the GeneTree topologies, fatty acid binding proteins (GeneTree IDs: 13715, 104992, 16199, 33149, 40763, 5377), appear to have expanded independently in *H. microstoma* and *S. erinaceieuropaei*. In the case of the galactosyltransferases, a considerable expansion is apparent in *S. erinaceieuropaei* within one particular branch (GeneTree ID: 1090).

**Table 3:** **Total protein length of gene families described as expanded in other tapeworm species**

**Family**	***E. granulosus***	***E. multilocularis***	***T. solium***	***H. microstoma***	***C. sinensis***	***S. mansoni***	***S. erinaceieuropaei***	**Expansion**
Heat shock protein, HSP70	22,013	23,205	17,547	2,884	7,460	4,100	4,441	No
Tetraspanin	8,547	9,268	8,253	6,514	2,442	3,683	3,742	No
Glycosylphosphatidylinositol (GPI)-anchored protein, GP50	2,326	8,441	4,604	5,347	0	0	0	No
Antigen B	590	780	329	428	0	0	0	No
EG95	306	626	1,350	0	0	0	0	No
Sporozoite threonine and asparagine-rich protein (STARP)-like antigen	1,519	2,072	2,517	772	1,661	1,783	388	No
Galactosyltransferase	6,685	8,690	5,689	2,937	1,264	2,157	10,296	Yes
Mu class glutathione S transferase	1,142	1,919	2,044	2,044	437	413	1,371	Yes
Fatty acid binding protein 2	799	586	670	1116	125	231	1,016	Yes

Protein family trees containing genes belonging to the tapeworm gene expansions described by Tsai *et al.* [2] were extracted from the EnsemblCompara GeneTrees database. The cumulative number of codons is described here for each species. The last column notes whether this family is likely to be expanded in all tapeworms.

A number of previously described antigen families were also apparently absent from *S. erinaceieuropaei* - EG95, Antigen B and GP50. There were proteins from *S. erinaceieuropaei* classed with the STARP-like antigen family, although they were few and noticeably absent from the predominant branch of this tree (GeneTree ID: 8926). These findings, across four antigen families, suggest it is quite likely that *S. erinaceieuropaei*, and perhaps the Diphyllobothriidea, do not, in general, share the same antigen family expansions as the Cyclophyllidea tapeworms.

The most expanded gene family encoded one group of dynein molecular motors. When we examined families inclusive of the other 15 *E. multilocularis* heavy chain dyneins annotated on GeneDB we found that the dynein motors in general were not expanded to the same degree (total length for *E. multilocularis* = 14,969, total length for *S. erinaceieuropaei* = 17,067, ratio of *S. erinaceieuropaei* to *E. multilocularis* = 1.14), indicating that this subset may have specific importance to *S. erinaceieuropaei*.

One of the top gene families (rank 5), consisting of a number of paralogs of FUT8, closest in sequence to alpha (1, 6) fucosyltransferases, was highly expanded in *S. erinaceieuropaei*. These enzymes have been shown to provide core fucosylation at N-glycans [36]. Glycosyltransferases, which add core 2 O-glycan branches (rank 76) and galactosyltransferase proteins (rank 8) were also expanded in *S. erinaceieuropaei*. These enzymes may create greater complexity at the protein structure level of glycoproteins in *S. erinaceieuropaei*. A number of other gene families involved in post-translational modification of proteins came up as expanded: several kinases, primarily serine/threonine kinase families and some proteins involved in protein folding (Kelch protein 18 and peptidylprolyl *cis-trans* isomerase 3).

We categorized each family into one of ten top level functions to further aid visual interpretation of the data: structural/cellular transport, regulation of transcription, post-translation modification or processing, transporter, receptor/signal transduction, protease, mRNA processing, metabolic processing/detoxification, cell cycle or DNA repair and unknown (Table 4). A large number of expansions contained proteins of unknown function. A BLASTX search of the *S. erinaceieuropaei* genes against the UniProt database [37] returned uncharacterised proteins with the following exceptions. All *S. erinaceieuropaei* genes within GeneTree 40097 returned hits to putative AMP-dependent ligases in *S. mansoni* (2 to 7, 9 and 11), known for their action in processing fatty acids. Genes within GeneTree 40961 returned hits to human Flt3-interacting zinc finger proteins (which interact with the receptor tyrosine kinase Flt3) and genes within GeneTree 66872 gave hits to *S. mansoni* putative rac guanyl-nucleotide exchange factor.

**Table 4:** **Summary of categorized gene family expansions**

**Top-level category**	**Number of expansions within category**
■ Unknown	20
♦ Regulation of transcription	14
❖ Structural/cellular transport	11
★ Post-translational modification or processing	9
✪ Metabolic processing/detoxification	9
Δ Transporter	6
✚ mRNA processing	5
↲ Receptor/signal transduction	3
○ Cell cycle or DNA repair	3
◉ Protease	2

Symbols cross-reference with those in Additional file 7.

Almost half of all gene families in our comparative analyses were unique to *S. erinaceieuropaei* (14,530 out of 22,026) - this large number may reflect clustering of partial components of genes. We took the 20 largest (in total protein length) of these unique gene families and investigated whether we could identify related proteins by BLASTX against the UniProt database [37]. The genes within these families did not return any significant hits to annotated proteins.

## Discussion

In this study, we report the third case of sparganosis in Europe, a cerebral infection with *S. erinaceieuropaei* in East Anglia, UK. After an initial biopsy failed to reveal the presence of the worm, and not knowing the cause of the lesion, we observed the migration pattern of the worm develop over four years, including its passage over to the opposite hemisphere of the brain. Using DNA extracted from the worm, the morphological diagnosis was refined to the species level, and the remainder of the sample was used to sequence and assemble the genome *de novo*. We investigated known and potential drug targets in the genome and all of the genome data are publicly available.

This case demonstrates the long-lived and active nature of a sparganosis larva in a human host, and how early diagnosis and recognition of this pattern would benefit future patients, minimizing tissue damage over critical regions of brain. The patient in this case suffered from a variety of neurological symptoms that changed in nature over the course of the infection. It is possible that some of these could have been prevented if the infection was recognized at an earlier stage. The case reported here occurred before publication of a study by Gong *et al*. [38] that focused on the MRI characteristics of 18 children diagnosed with cerebral sparganosis. In the eight children that had MRI scan data over time, migration of lesions was observed in three. Gong *et al*. also reported on the different MRI enhancement patterns observed, which included ring-enhancing lesions similar to those observed in this patient, half of which were characterized as beaded or nodular. Here we also observe the presence of multiloculate lesions. Therefore, in future cases, when other more common potential causes (such as tuberculosis) are ruled out, a migration pattern with ring-enhancing lesions, particularly multiloculate, should raise suspicion of sparganosis.

Sparganosis is a general term for infection with a subclass of tapeworms, as the different species that can be responsible are not distinguishable by eye. However, the exact species of worm can affect the prognosis for the patient. *S. erinaceieuropaei* is the more common causative agent. *S. proliferum* is the most mysterious of the sparganosis-causing worms, as its adult form has never been observed. The defining characteristic of *S. proliferum* is its ability to proliferate in the host, and it has also been defined as a separate species at the molecular level [39]. It is exceptionally rare but has been observed in a number of cases that have proved fatal. Determining the species of worm as *S. erinaceieuropaei* in this infection, based on its mitochondrial cytochrome oxidase 1 sequence, was therefore positive news for the patient in this case.

Identifying the species at the molecular level also gives us a clue as to the origin of infection. *S. mansonoides* is reported as the *Spirometra* species found in the Americas, whilst *S. erinaceieuropaei* is the species more commonly reported in East Asia. A population genetics study of *S. erinaceieuropaei* was previously conducted in Hunan province, China utilising two other mitochondrial genes, *nad1* and *cox3* [9]. In order to investigate the geographical origin we also sequenced these markers and found that both were identical to some of the haplotypes found in the previous study. The fact that in just one provincial population polymorphism is seen in these genes, and that we found sequences that were identical to some of these, suggests that the infection originated in China. This is consistent with the patient’s travel history.

With an increase in global mobility, infections such as sparganosis that have previously been constricted to a certain region may increasingly appear in places with no prior history [40]. Recording such events and sharing molecular data will be critical for a greater understanding of the epidemiology of infections and to help clinicians understand the potential diagnoses in their geographical area.

Previously there has been a paucity of molecular data for *S. erinaceieuropaei*; reports in the literature have focused on the mitochondrion [41]; a small number of cloned nuclear genes, such as genes encoding copper/zinc-superoxide dismutase [42] and a ced-3-like apoptosis-related gene [43]; and a survey of 910 ESTs [44]. Recently, the genomes of four different species of tapeworm were described [2,3] but, for the first time, a genome from the Diphyllobothriidea order of tapeworms is now available. This genome will not only enable insights into *S. erinaceieuropaei* but also into other species of the group, including the important fish parasites of *Diphyllobothrium spp*. [16].

At 1.26 Gb, the present sequence is the largest reported for a flatworm. In particular, it is nearly 10 times larger than the genomes of the published cyclophylid tapeworms (which range from 115 to 152 Mb) [2,3]. Some of this size difference is likely to be due to the fragmentary nature of the assembly. Assesment of read depth in mapped sequencing data suggests that the potential contribution of split alleles to the genome size is low. The *S. erinaceieuropaei* predicted proteome (68.4 Mb) is only somewhat larger than those of other tapeworms (50.7 Mb in *E. multilocularis* and 46.4 Mb in *Hymenolepis microstoma*) and indeed comparable to the proteome of the trematode *S. mansoni* (68.2 Mb); therefore, artefactual duplications in the assembly are unlikely to account for its huge genome size. Longer introns, which average 1,065 bp in comparison with 573 to 863 bp in the Cyclophyllidea species, may inflate the genome. In addition, the genome is much more repetitive than that sequenced from other tapeworms; almost half of the *S. erinaceieuropaei* genome size is apportioned to repetitive elements - much greater than in sequenced Cyclophyllidea species (7 to 11%) [2]. Of these elements, LINEs constitute a large percentage, in contrast to cyclophylids, which have far fewer.

Our initial approach to interrogating the genome concentrated on the targets of current tapeworm chemotherapy, and on candidate novel targets identified from genome data. The gene for the most highly expressed β-tubulin in the larval stage of *E. multilocularis* (EmuJ_000672200, or *tub-2)* contains resistance-associated amino acids. It has been suggested that this accounts for the reduced sensitivity of the cestode larval stage to benzimidazole drugs [17]. We found an *S. erinaceieuropaei* orthologue, which we predict to be insensitive to albendazole based on the presence of tyrosine amino acid residues in positions that are known to confer resistance in other organisms. We reasoned that if the orthologue expression pattern is similar across species [45], then, as with *E. multilocularis*, benzimidazole would likely be suboptimal for chemotherapy against larval tapeworms of *Spirometra*. Using recently published EST data from the larva of *S. erinaceieuropaei* [20], we identified β-tubulin transcripts and found that the majority contained the benzimidazole resistance-associated amino acids.

Cases of sparganosis that were unresponsive to praziquantel have previously been reported [46]. Both sensitive and resistant configurations of a proposed target of praziquantel, CaV2 B, are encoded by the genome. Future studies addressing the mode of action of praziquantel and target protein amino acid dependencies, along with functional studies of tapeworms, may reveal the underlying genetic basis of reported resistance. The greater number of ATP cassette domains identified in *S. erinaceieuropaei* gene transcripts in comparison with *E. multilocularis* may indicate a greater number of functional genes, with perhaps greater diversity in the worm’s efflux capabilities and therefore its ability to process xenobiotic compounds.

As new drugs against tapeworms are introduced, shared molecular targets, some putative examples of which are summarized in our results, can continue to be assessed using genome level information on *S. erinaceieuropaei.* In terms of suitable drug action, in cerebral cases even drugs that prevent movement of the worm (and hence more widespread tissue disruption) could be beneficial if curative surgery is delayed or not possible because of patient health or the location of the worm. In cases that affect the central nervous system, such as in the presented case, the associated side effects of any drug treatment should also be considered. In our study we also identify proteins that are likely to be involved in host-parasite interactions, which may feed into treatment considerations or possible new diagnostic tests (for example, a serological reaction against recombinant PGF). In the present case, inflammation in the brain in response to the worm is likely to have contributed to the patient’s symptoms; determining whether or how the live worm modulates inflammation could provide vital information for choosing between drug treatment or surgery.

We also observed expansions in serine proteases and Kunitz-type protease inhibitors in *S. erinaceieuropaei* compared with *E. multilocularis* and *E. granulosus*, which may aid *S. erinaceieuropaei* in its invasion of a wide range of hosts. Interestingly, chymotrypsin A-like proteases were the most expanded serine protease class. Within nematodes, a large expansion of this class was also described in *T. muris*, which lives in close association with the host gut [31]. Here, therefore, we may be observing convergent utilisation of this set of proteases in two unrelated parasites.

We used the genome to examine expanded gene families in *S. erinaceieuropaei*. Nine out of the 25 most frequently expressed Pfam domains reported in *S. erinaceieuropaei* EST data [20] are also present in the top expanded gene families that we have identified. Thus, expanded gene families (protein kinase, BTB/Kelch associated, EF hand, WD40 repeat, Kelch motif, fibronectin type III, zinc finger C2H2, AMP-dependent synthetase and dynein light chain) are also amongst the most expressed and therefore likely to be functionally important to the organism. Nine expanded families appear to be involved in transcriptional regulation. The life cycle of *S. erinaceieuropaei* is composed of discrete morphologically distinct multicellular forms adapted for different hosts. Therefore, a complex set of transcriptional regulators would be expected to coordinate the expression of proteins required for each stage. A further nine expanded gene families appear to be associated with metabolic processing or detoxification pathways. It is possible that a range of metabolic and detoxification adaptations allow the parasite to live in a wide range of hosts (crustaceans, reptiles, amphibians and mammals) as well as in aquatic environments, as is the case for the free-swimming miracidia. The 20 expanded gene families with unknown function demonstrate how little we know about this order of tapeworms.

As sparganosis is a rare infection, drug re-purposing may offer the greatest hope for the patients afflicted. In terms of new potential targets for intervention, in *S. erinaceieuropaei* we observed the largest diversity of metalloproteases of the M17 class reported in any organism thus far. Leucyl aminopeptidases of the M17 class have been considered potential targets for antimalarial drugs [47,48] and with active drug discovery programmes underway [49] new open access drugs will be developed for malaria that could be used against more neglected parasites. Publicly available genome level information on *S. erinaceieuropaei*, and its continual interrogation by the medical research community, will facilitate the necessary inferences to be made concerning the cross-applicability of the latest chemotherapy treatments.

## Conclusions

We have reported the first known case of sparganosis in the UK and have diagnosed the infective species to be the pseudophyllidean tapeworm *S. erinaceieuropaei*, using DNA isolated from a surgical biopsy. Previously, sparganosis has predominantly been reported in Asia and this case highlights how an increase in global mobility can bring new challenges to clinicians facing infections from outside their usual geographical range. By describing the clinical presentation, in which a multiloculate lesion was seen migrating across the brain, we hope that this rare but debilitating infection will be on the radar as a diagnostic possibility for future cases.

Given the paucity of molecular data for this human pathogen, we used the small quantity of DNA present in a biopsy sample to generate a genome *de novo*.

The genome represents the first draft genome from the order Diphyllobothriidea. Aware of the fragmented nature of the assembly, we have conservatively analysed its gene content, in the context of comparisons with other flatworms, and found a diverse set of gene expansions that are not present in other tapeworms previously sequenced. These include genes that may be key to the organism’s success in multiple divergent hosts and tissue types.

From the genome data we have evaluated potential druggability and our results suggest that albendazole is unlikely to be effective but that many drugs previously proposed as candidates for repurposing against more common tapeworms are likely to also be effective against *S. erinaceieuropaei.* The availability of the genome data will provide an ongoing reference for similar molecular comparisons.

## Materials and methods

### Ethics statement

The patient has given written consent allowing for publication of this case and associated images. To remove any patient data from our reference genome, sequencing reads were screened against the human 1000 genome reference assembly, NCBI36, [50] using the Burrows-Wheeler Aligner software package (aln and sampe command) with default settings [51]. The forward and reverse reads were aligned independently and any matches were removed, along with the paired read, to a separate file with permissions that deny access.

### Pathology/histology methods

The neurosurgical specimen was formalin-fixed and processed to paraffin for sectioning (5 micron thickness). Haematoxylin and eosin (H&E), PAS, Grocott methenamine silver, Ziehl-Nielsen and modified Ziehl-Nielsen stains were applied. Inflammatory infiltrates were immunocytochemically stained with commercially available antibodies to CD3 (NovoCastra, Newcastle upon Tyne, Tyne and Wear, UK), CD79a (Dako, Glostrup, Hovedstaden, Denmark) and CD68 (Dako) for T cells, B cells and microglia and macrophages, respectively. For images a Leica DMLB microscope with Leica DFC320 digital camera was used in conjunction with Leica IM50 Image Manager Version 4.0 software (Leica Microsystems Imaging Solutions Ltd, Cambridge, UK).

### DNA extraction

A slide-mounted unstained section of worm was manually detached from substrate using an adjacent stained sample as a guide. The worm sample was then deparaffinized and the DNA extracted using the QIAamp DNA FFPE Tissue Kit (Qiagen, Venlo, Limburg, Netherlands). DNA was measured using Qubit® fluorometric quantification (97 ng total).

### Molecular diagnosis

PCR was carried out using primers for the mitochondrial cytochrome oxidase c subunit 1 (*cox1*) as used by Liu *et al.* [14]: JB3 5′-TTTTTTGGGCATCCTGAGGTTTAT-3′, JB4 5′-TAAAGAAAGAACATAATGAAAATG-3′. PCR was also carried out using primers for *nad1* (Senad1F 5′-ATAAGGTGGGGGTGATGGGGTTG-3′, Senad1R 5′- ATAAAAAATAAAAGATGAAAGGG-3′) and *cox3* (Secox3F 5′- GGGTGTCATTTCTTCCTATTTTTAA-3′, Secox3R 5′- AAATGTCAATACCAAGTAACTAAAG-3′), as described in Liu *et al*. [52]. PCRs (50 μl) were performed in 1× KAPA HiFi HotStart ReadyMix (Kapa Biosystems, Wilmington, MA, USA) with 50 pmol of each primer and 1 μl sample (0.485 ng/μl). Reaction conditions were an initial denaturation at 98°C for 5 minutes, followed by 35 cycles of 98°C for 20 s, 55°C for 15 s, 72°C for 30 s, then a final extension step of 72°C for 5 minutes. After gel electrophoresis, bands were cut out from the agarose and extracted using the QIAquick® Gel Extraction Kit (Qiagen). The DNA was capillary sequenced at the Wellcome Trust Sanger Institute using SP6 and T7 sequencing primers. A high quality consensus sequence from both reads was used for analysis.

### Paired-end illumina sequencing

DNA (48.5 ng) was used for the preparation of a paired-end Illumina library. Briefly, DNA was fragmented to 400 to 550 bp using Adaptive Focused Acoustics technology with the E210 instrument (Covaris, Woburn, MA, USA) (duty cycle 20; intensity 5; cycles/bursts 200; seconds 30; temperature 4°C). After the DNA was fragmented it was cleaned and concentrated with a 1:1 ratio of Ampure XP magnetic beads. This was repeated after subsequent end repair and DA-tailing reactions with the respective modules supplied by New England Biolabs (Ipswich, MA, USA) (NEBNext™ DNA Sample Prep Reagent Set 1: E6000), following the manufacturer’s instructions. To ligate sequencing adaptors, a 50 μl reaction mixture containing the sample was set with addition of 25 μl of 2× DNA T4 ligase buffer (New England Biolabs, Inc.), 4 μl 4 μM Illumina paired-end duplex adaptors (Integrated DNA Technologies, Coralville, IA, USA) and 2 μl T4 DNA ligase. The ligation reaction was incubated at 20°C for 30 minutes before a 1:1 ratio round of clean up, with Ampure XP magnetic beads. This was then repeated with a 0.7:1 ratio of beads to sample to remove adaptor dimers. Eight cycles of PCR were carried out on the sample using 1× KAPA HiFi HotStart ReadyMix (Kapa Biosystems) with paired-end primers 1.0 and 2.0 (Ilumina). The resulting library was loaded for a paired-end sequencing run on the Illumina HiSeq 2000 system with 100 cycles. This generated 54,723,550,600 bp of data, representing approximately 43× coverage.

### *De novo* genome assembly

Short paired-end sequence reads were first corrected and initially assembled using SGA v0.9.7 [53]. The distribution of k-mers for all odd values of k between 41 and 81 was calculated using GenomeTools v.1.3.7 [54]. A k-mer length of 75, selected as the length that produced the maximum number of unique k-mers, was used for de Bruijn graph construction in a subsequent assembly with Velvet v1.2.03 [55]. Approximately 1,103 CPU hours were used for assembly, with a peak memory usage of 116 GB.

### Genome assembly quality assessment

When mapped back to the assembly with SMALT, raw sequencing data from each lane (lane 8823_7 and lane 9489_2) gave a peak insert size of 400 to 450 bp (Additional file 8) and a low duplicate rate of 8.3% and 8.8%, respectively. The percentage of rble as assessed using eads containing low quality sequence or adaptor sequence was negligible as assessed using Trimmomatic [56] (3.32%). REAPR detects possible misassembly sites using paired-end reads and then breaks the assembly to give the most conservative but accurate representation of the assembly [57]. We found that after using REAPR the N50 only decreased by approximately 100 bp from 4.6 to 4.5 kb, with 12,687 extra scaffolds, whilst the largest scaffold remained the same. To investigate the potential for collapsed regions or split alleles in the genome, we examined coverage of a subset of SMALT mapped data (lane 882_7) across 5-kb binned regions in scaffolds that were 6 kb or longer. The mean coverage was 16.9 with a median of 15.4 (interquartile range 6.72). We found that 7% of the genome was below 0.6× median coverage, and 8% was above 1.6× median coverage. For the mitochondrial genome, we found that 137 contigs in a BLAST search against the mitochondrial sequence of a Chinese isolate [41] gave a significant match with an E value of <1e-50.

### Gene predictions

Gene prediction for *S. erinaceieuropaei* was conducted by various methods available in MAKER version 2.2.28 [15]. The MAKER annotation pipeline consists of four general steps to generate high-quality annotations by taking into account evidence from multiple sources. First, assembled contigs are filtered against RepeatRunner [58] and a species specific repeat library (generated by RepeatModeler [18]) using RepeatMasker [19] to identify and mask repetitive elements in the genome. Second, gene predictors Augustus 2.5.5 [59], GeneMark-ES 2.3a (self-trained) [60] and SNAP 2013-02-16 [61] are employed to generate *ab initio* gene predictions that can use evidence within MAKER. Further species-specific gene models were provided to MAKER using comparative algorithms against the *S. erinaceieuropaei* genome: genBlastG [62] output of *C. elegans* gene models from Wormbase [63] and RATT [64] output of *H. microstoma* gene models [2]. These models cannot be influenced by MAKER evidence as they were provided by gff file. Next, species-specific cDNAs available from the International Nucleotide Sequence Database Consortium [65] and proteins from related organisms were aligned against the genome using BLASTN and BLASTX [66], and these alignments were further refined with respect to splice sites using Exonerate [67]. Finally, the protein homology alignments, comparative gene models and *ab initio* gene predictions are integrated and filtered by MAKER and project specific scripts to produce a set of evidence-informed gene annotations.

The MAKER genome annotation pipeline was run three consecutive times. In the absence of a species-specific trained gene predictor, Augustus and SNAP were trained using CEGMA [68] protein evidence gained from the default KOGs and hidden Markov model profiles of Cestode orthologous groups (CEOGs; unpublished by MM and JM). The first run of MAKER was performed using the est2genome and protein2genome option with the handful of taxonomy-specific cDNAs, and platyhelminth protein sequences, respectively. Gene models obtained from the first run were used to retrain SNAP and models from the second run were used to retrain Augustus. With the trained models, MAKER was run a third time using a taxonomically broader protein set that included metazoan proteins from the UniProt Complete protein database [37] and a subset of helminth proteomes from GeneDB [69].

### Comparative analysis

The InterProScan 5 tool was used to provide domain-level predictions on predicted gene transcripts [70]. Protease and protease inhibitors were characterized using the specialist database MEROPS [34]. InterPro domains with the keywords protease, proteinase, proteolytic or peptidase were used to obtain the geneIDs and subsequently the transcript FASTA files for candidates. Candidate transcript sequences were submitted as a batch BLAST to MEROPS, which provided a report on protease family hits.

EnsemblCompara GeneTrees (v75) is a fault-tolerant pipeline to run orthology and paralogy gene prediction analysis using TreeFam methodology to provide a complete set of phylogenetic trees [71]. The Cestoda species included in the comparison with *S. erinaceieuropaei* were *E. multilocularis*, *E. granulosus*, *T. solium* and *H. microstoma. Trematoda* species *S. mansoni* and *C. sinensis* were also included in the comparison. Outgroups included were *C. teleta* and *C. gigas*. International Nucleotide Sequence Database Collaboration (INSDC) genome assemblies and project IDs for ComparaEnsembl comparative analysis were as follows: *C. teleta*, Capca1 (PRJNA175705); *C. gigas*, oyster_v9 (PRJNA70283); *T. solium*, TSMEXv1 (PRJNA170813); *E. granulosus*, EGRAN001 (PRJEB121); *E. multilocularis*, EMULTI001 (PRJEB122); *H. microstoma*, HMIC001 (PRJEB124); *S. mansoni*, ASM23792v2 (PRJEA36577); *C. sinensis*, C_sinensis-2.0 (PRJDA72781). For each species considered in the analysis, the longest protein translation for each gene is identified. Each protein is queried using NCBI-BLAST against each individual protein within (self-species) and between all species [72]. From these results graphs are constructed. Connections (edges) between the nodes (proteins) are retained when they satisfy either a best reciprocal hit (BRH) or a BLAST score ratio (BSR) over 0.33. From the graph, the connected components (that is, single linkage clusters) are extracted. Each connected component represents a cluster, that is, a gene family. If the cluster has greater than 750 members, the graph construction and clustering steps are repeated at higher stringency. Proteins in the same cluster are aligned using MUSCLE to obtain a multiple alignment [73]. The coding sequence back-translated protein-based multiple alignment is used as an input to the tree program, TreeBeST, as well as a multifurcated species tree which is necessary for reconciliation and the duplication calls on internal nodes [74]. The resulting trees are flattened into ortholog and paralog tables of pairwise relationships between genes. In the case of paralogs, this flattening also records the timing of the duplication due to the presence of extant species past the duplication, and thus implicitly outgroup lineages before the duplication. This method produces trees with less anomalous topologies than single protein-based phylogenetic methods.

### Data availability

Sequences for *cox3* and *nad1* amplicons from the clinical sample have been deposited in GenBank under accession IDs KM031786 and KM031787, respectively. The *S. erinaceieuropaei* genome, predicted transcripts, protein and annotation (*.GFF) files are available from the Wormbase resource [63] under BioProject PRJEB1202 (S_erinaceieuopaei_v1_0_4) [75].

Accession numbers LN000001 to LN482396 in the European Nucleotide Archive (ENA) cover the *S. erinaceieuropaei* genome assembly. The raw data (Illumina reads) are available from ENA via accession number ERS182798. ComparaEnsembl GeneTree IDs and tree in Newick format are available in Additional file 3.

Parasite genome assemblies used in the ComparaEnsembl GeneTree analysis are available through the Wormbase resource with the following BioProject IDs and version names: *E. multilocularis*, PRJEB122 (EMULTI001); *E. granulosus*, PRJEB121 (EGRAN001); *H. microstoma*, PRJEB124 (HMIC001); *S. mansoni*, PRJEA36577 (ASM23792v2); *C. sinensis*, PRJDA72781 (C_sinensis-2.0). Outgroup genomes are available from INSDC: *C. teleta*, PRJNA175705 (Capca1); *C. gigas*, PRJNA70283 (oyster_v9).

## Additional files

Additional file 1:
**Axial MRI images from patient before surgery (2012).** T1 weighted scan before (C) and after (D) the injection of gadolinium. Gadolinium multiple ring enhancing lesions are present in the left thalamic region and adjacent posterior limb of the internal capsule (D). T2 weighted scan shows an area of brain oedema (A) surrounding the lesions. Diffusion weighted imaging showed no restriction in diffusion (B), indicating that the lesion was not recently ischaemic. Additional file 2:
***Cox1*** **in genome data.** The *cox1* amplicon aligned with the best genome hit from BLAST; asterisks indicate consensus sequence and numbers on the right-hand side indicate position within each sequence. Base differences from previously reported *S. erinaceieuropaei cox1* sequence are confirmed in the genome. The genome matches previously reported *S. erinaceieuropaei cox1* sequence at two sites at the tail of the amplicon sequencing where bases differ in the amplicon. Additional file 3:
**ComparaEnsembl GeneTree IDs and trees.** GeneTree IDs and corresponding trees generated from the ComparaEnsembl clustering pipeline. Additional file 4:
**Table of gene expansions by number of genes in family.** *E. multilocularis* was included because it has the most complete reference genome of the tapeworms. This table shows a list of the most obvious expanded gene families, exhibiting at least 10 times as many genes as in *E. multilocularis.* This criterion gave 39 expanded families in *S. erinaceieuropaei*. Annotation and description of biological function were provided by examining the annotation for the *E. multilocularis* and *S. mansoni* members of each GeneTree. Additional file 5:
**Distribution of median protein length of predicted** ***Cestoda*** **gene families.** Histogram showing the distribution of the median protein length for each species, within each family predicted by the EnsemblCompara GeneTree pipeline. Median protein length has a more compact distribution in *S. erinaceieuropaei* than the other species, and drops comparatively sharply after 300 amino acids. Additional file 6:
**Scatterplot of the** ***E. multilocularis*** **median protein length in each EnsemblCompara GeneTree family against the number of** ***S. erinaceieuropaei*** **proteins predicted in the family.** The function of the line drawn is Y = 0.002324 × X + 0.686443, with a *P*-value of <2e-16 and an adjusted R^2^ value of 0.1648. A large amount of biological variation is likely to explain the lack of points contributing to this linear model. Note, however, that the linear model does extrapolate to titin, the largest known natural protein, which artificially appears expanded in *S. erinaceieuropaei*. The total predicted protein length of the titin family in *E. multilocularis* is 11,194 amino acids, which is greater than the 9,086 amino acids in *S. erinaceieuropaei*. Additional file 7:
**Gene families obtained from the EnsemblCompara GeneTrees pipeline ranked by the ratio of the cumulative length of proteins in the gene family of** ***S. erinaceieuropaei*** **to** ***E. multilocularis*** **.** The criteria for expansion was set at 3 and above, resulting in 83 gene families for further investigation. The table contains the consensus indicative annotation for *E. multilocularis* and *S. mansoni* members of the gene family, a brief description of the associated biological function (as ascertained by the supported text in UniProt, NCBI and Pfam) and an assignment to one of ten top-level categories. These catergories are: structural/cellular transport (❖), regulation of transcription (♦), post-translation modification or processing (★), transporter (Δ), receptor/signal transduction (↲), protease (◉), mRNA processing (✚), metabolic processing/detoxification (✪), cell cycle or DNA repair (○) and unknown (■). Additional file 8:
**Insert size of two lanes of paired-end Illumina HiSeq 2000 data. Raw reads for each sequencing lane were mapped back to the genome assembly using SMALT to determine the insert size distribution.** Both data sets were within the required range.

## Abbreviations

bp: base pair
CEGMA: Core Eukaryotic Genes Mapping Approach
EST: expressed sequence tag
INSDC: International Nucleotide Sequence Database Collaboration
LINE: long interspersed element
MRI: magnetic resonance imaging
PCR: polymerase chain reaction
PGF: plerocercoid growth factor
