# Supplementary figures and images for: The genome of the sparganosis tapeworm Spirometra erinaceieuropaei isolated from the biopsy of a migrating brain lesion
Source: Genome Biol. 2014 Nov 21;15(11):510. doi: 10.1186/s13059-014-0510-3 (PMC4265353; doi:10.1186/s13059-014-0510-3)

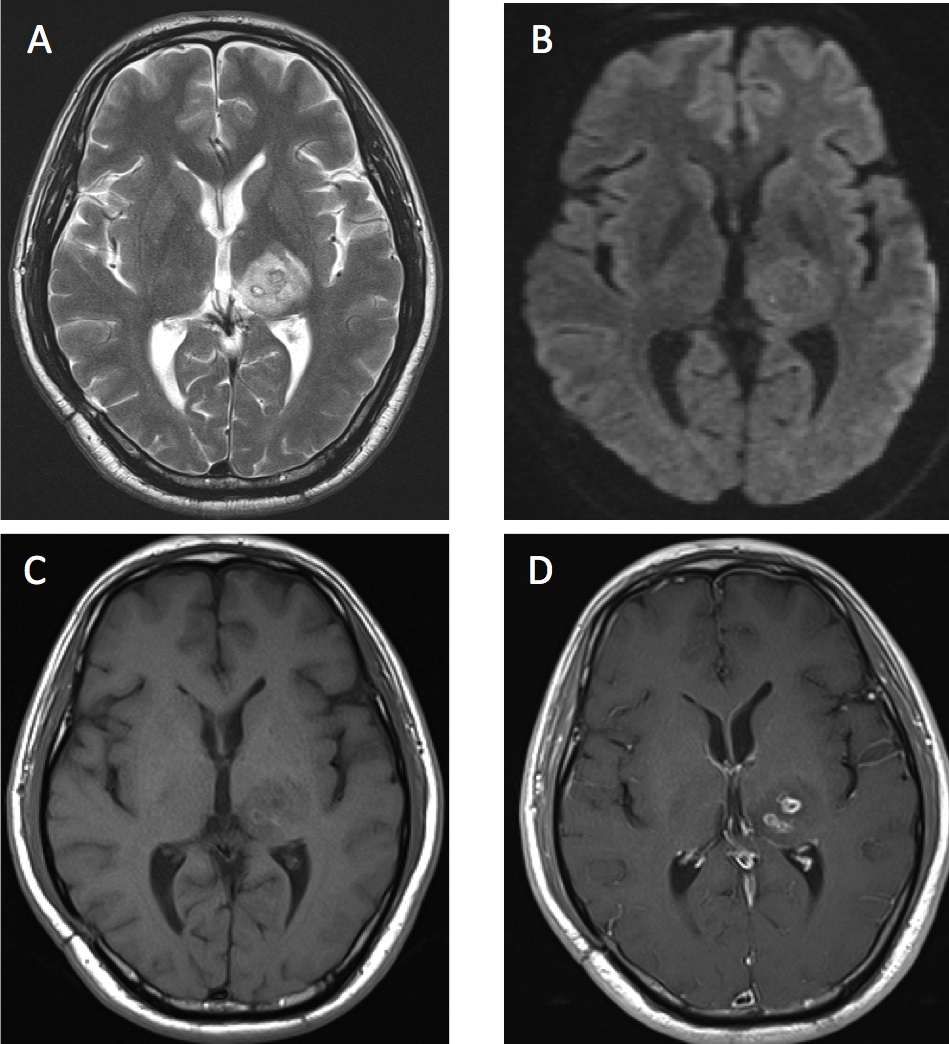

Supplement: Additional file 1: — Axial MRI images from patient before surgery (2012). T1 weighted scan before (C) and after (D) the injection of gadolinium. Gadolinium multiple ring enhancing lesions are present in the left thalamic region and adjacent posterior limb of the internal capsule (D). T2 weighted scan shows an area of brain oedema (A) surrounding the lesions. Diffusion weighted imaging showed no restriction in diffusion (B), indicating that the lesion was not recently ischaemic. [file 13059_2014_510_MOESM1_ESM.jpeg]

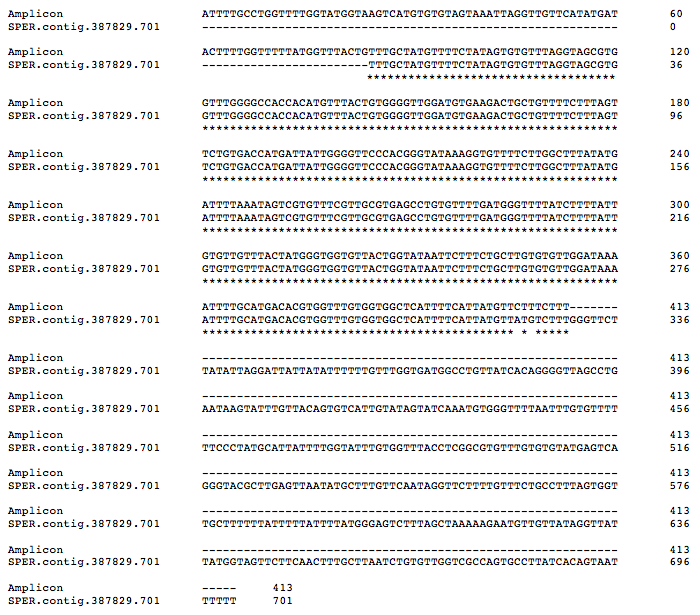

Supplement: Additional file 2: — Cox1 in genome data. The cox1 amplicon aligned with the best genome hit from BLAST; asterisks indicate consensus sequence and numbers on the right-hand side indicate position within each sequence. Base differences from previously reported S. erinaceieuropaei cox1 sequence are confirmed in the genome. The genome matches previously reported S. erinaceieuropaei cox1 sequence at two sites at the tail of the amplicon sequencing where bases differ in the amplicon. [file 13059_2014_510_MOESM2_ESM.png]

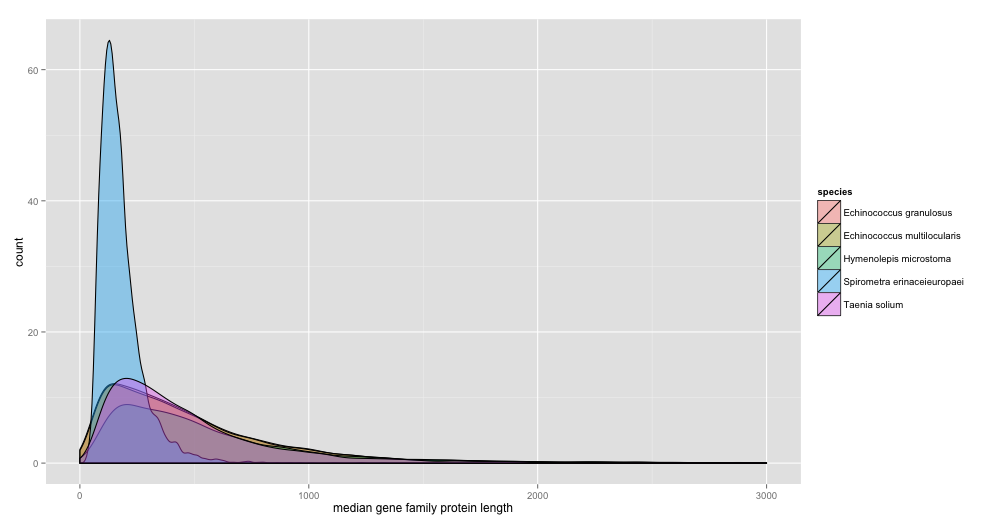

Supplement: Additional file 5: — Distribution of median protein length of predicted Cestoda gene families. Histogram showing the distribution of the median protein length for each species, within each family predicted by the EnsemblCompara GeneTree pipeline. Median protein length has a more compact distribution in S. erinaceieuropaei than the other species, and drops comparatively sharply after 300 amino acids. [file 13059_2014_510_MOESM5_ESM.tiff]

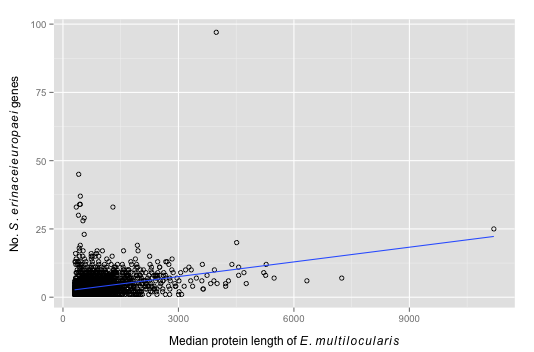

Supplement: Additional file 6: — Scatterplot of the E. multilocularis median protein length in each EnsemblCompara GeneTree family against the number of S. erinaceieuropaei proteins predicted in the family. The function of the line drawn is Y = 0.002324 × X + 0.686443, with a P-value of <2e-16 and an adjusted R2 value of 0.1648. A large amount of biological variation is likely to explain the lack of points contributing to this linear model. Note, however, that the linear model does extrapolate to titin, the largest known natural protein, which artificially appears expanded in S. erinaceieuropaei. The total predicted protein length of the titin family in E. multilocularis is 11,194 amino acids, which is greater than the 9,086 amino acids in S. erinaceieuropaei. [file 13059_2014_510_MOESM6_ESM.tiff]

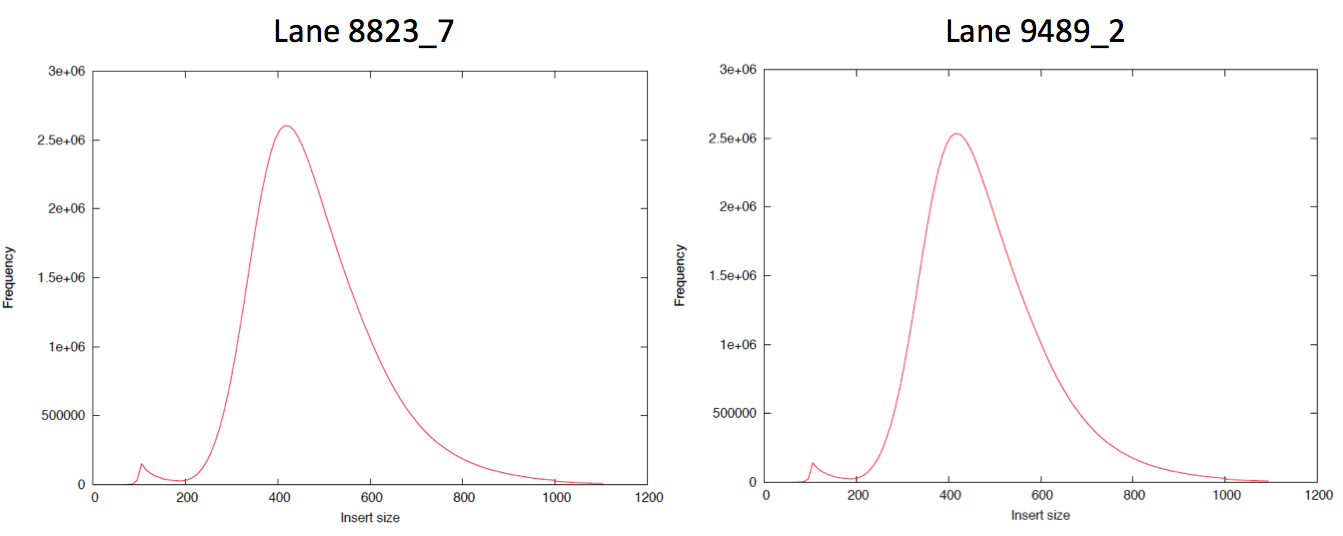

Supplement: Additional file 8: — Insert size of two lanes of paired-end Illumina HiSeq 2000 data. Raw reads for each sequencing lane were mapped back to the genome assembly using SMALT to determine the insert size distribution. Both data sets were within the required range. [file 13059_2014_510_MOESM8_ESM.tiff]
